# Cardiorespiratory Fitness Moderates the Age-Related Association Between Executive Functioning and Mobility: Evidence From Remote Assessments

**DOI:** 10.1093/geroni/igac077

**Published:** 2022-12-22

**Authors:** Emma Gabrielle Dupuy, Florent Besnier, Christine Gagnon, Juliana Breton, Thomas Vincent, Catherine-Alexandra Grégoire, Catia Lecchino, Marie Payer, Béatrice Bérubé, Miloudza Olmand, Marianne Levesque, Nadia Bouabdallaoui, Josep Iglesies-Grau, Martin Juneau, Paolo Vitali, Mathieu Gayda, Anil Nigam, Louis Bherer

**Affiliations:** Centre ÉPIC and Research Center, Montreal Heart Institute, Montreal, Quebec, Canada; Department of Medicine, Université de Montréal, Montreal, Quebec, Canada; Centre ÉPIC and Research Center, Montreal Heart Institute, Montreal, Quebec, Canada; Department of Medicine, Université de Montréal, Montreal, Quebec, Canada; Centre ÉPIC and Research Center, Montreal Heart Institute, Montreal, Quebec, Canada; Centre ÉPIC and Research Center, Montreal Heart Institute, Montreal, Quebec, Canada; Centre ÉPIC and Research Center, Montreal Heart Institute, Montreal, Quebec, Canada; Centre ÉPIC and Research Center, Montreal Heart Institute, Montreal, Quebec, Canada; Centre ÉPIC and Research Center, Montreal Heart Institute, Montreal, Quebec, Canada; Department of Psychology, Université de Montréal, Montreal, Quebec, Canada; Centre ÉPIC and Research Center, Montreal Heart Institute, Montreal, Quebec, Canada; Department of Psychology, Université du Québec à Montréal, Montreal, Quebec, Canada; Centre ÉPIC and Research Center, Montreal Heart Institute, Montreal, Quebec, Canada; Department of Psychology, Université du Québec à Montréal, Montreal, Quebec, Canada; Centre ÉPIC and Research Center, Montreal Heart Institute, Montreal, Quebec, Canada; Department of Psychology, Université de Montréal, Montreal, Quebec, Canada; Centre ÉPIC and Research Center, Montreal Heart Institute, Montreal, Quebec, Canada; Department of Psychology, Université de Montréal, Montreal, Quebec, Canada; Centre ÉPIC and Research Center, Montreal Heart Institute, Montreal, Quebec, Canada; Department of Medicine, Université de Montréal, Montreal, Quebec, Canada; Centre ÉPIC and Research Center, Montreal Heart Institute, Montreal, Quebec, Canada; Department of Medicine, Université de Montréal, Montreal, Quebec, Canada; Centre ÉPIC and Research Center, Montreal Heart Institute, Montreal, Quebec, Canada; Department of Medicine, Université de Montréal, Montreal, Quebec, Canada; Department of Neurology and Neurosurgery, McGill University, Montreal, Quebec, Canada; Centre ÉPIC and Research Center, Montreal Heart Institute, Montreal, Quebec, Canada; Department of Medicine, Université de Montréal, Montreal, Quebec, Canada; Centre ÉPIC and Research Center, Montreal Heart Institute, Montreal, Quebec, Canada; Department of Medicine, Université de Montréal, Montreal, Quebec, Canada; Centre ÉPIC and Research Center, Montreal Heart Institute, Montreal, Quebec, Canada; Department of Medicine, Université de Montréal, Montreal, Quebec, Canada

**Keywords:** Cognition, Functional mobility, Moderation analysis, Physical fitness, Timed Up and Go

## Abstract

**Background and Objectives:**

In older adults, executive functions are important for daily-life function and mobility. Evidence suggests that the relationship between cognition and mobility is dynamic and could vary according to individual factors, but whether cardiorespiratory fitness reduces the age-related increase of interdependence between mobility and cognition remains unexplored.

**Research Design and Methods:**

One hundred eighty-nine participants (aged 50–87) were divided into 3 groups according to their age: middle-aged (MA; <65), young older adults (YOA; 65–74), and old older adults (OOA; ≥75). Participants performed Timed Up and Go and executive functioning assessments (Oral Trail Making Test and Phonologic verbal fluency) remotely by videoconference. Participants completed the Matthews questionnaire to estimate their cardiorespiratory fitness (VO2 max in ml/min/kg). A 3-way moderation was used to address whether cardiorespiratory fitness interacts with age to moderate the relationship between cognition and mobility.

**Results:**

Results showed that the cardiorespiratory fitness × age interaction moderated the association between executive functioning and mobility (β = −0.05; *p* = .048; *R*^2^ = 17.6; *p* < .001). At lower levels of physical fitness (<19.16 ml/min/kg), executive functioning significantly influenced YOA’s mobility (β = −0.48, *p* = .004) and to a greater extent OOA’s mobility (β = −0.96, *p* = .002).

**Discussion and Implications:**

Our results support the idea of a dynamic relationship between mobility and executive functioning during aging and suggest that physical fitness could play a significant role in reducing their interdependency.

**Translational Significance:** Preserving autonomy during aging is a major challenge of gerontology. This cross-sectional study focuses on functional mobility, using the Timed Up and Go, and demonstrates the moderating role of cardiorespiratory fitness in the age-related increase of cognitive–motor interdependency. Identifying the factors likely to affect older adults’ mobility, and understanding their interactions, may help tailor the preventive health interventions to the specific needs of each older adult. Performed in a coronavirus disease 2019 pandemic context, remote methods developed for this study are likely to support the development of research with isolated and rural geriatric populations underrepresented in clinical trials.

## Background and Objectives

Mobility restrictions gradually occur during the aging process and affect older adults’ ability to independently conduct their activities of daily living. If the role of physical declines, including cardiorespiratory and neuromuscular aging, has been widely documented to explain decreased mobility with aging (Fiser et al., 2010; Hunter et al., 2016; McGregor et al., 2014), more recent research also sheds light on the role of age-related cognitive decline. Indeed, a growing body of literature puts forward the close relationship between cognitive functioning and mobility in older adults, suggesting a crosstalk between these functions throughout aging (Rosano et al., 2005; Tian et al., 2017).

Behavioral and neuroimaging evidence suggests that older adults’ functional mobility is associated with their cognitive capacity (Li et al., 2018). In 2016, a meta-analysis based on 26 studies highlighted that older adults’ gross motor skills are broadly associated with fluid aspects of cognition (Demnitz et al., 2016). The most substantial relationship between mobility and cognition was reported for executive control mechanisms, which could even mediate age-related changes observed in mobility (Best et al., 2016; Clouston et al., 2013; Langeard et al., 2019). Interestingly, age appears to be a moderator of the cognitive–motor relationship; its effect would be minimal in middle-aged individuals, then would gradually increase after 65 years (Demnitz et al., 2018). Although its exact nature is still a matter of debate, emergent hypotheses suggest that this phenomenon could result from the age-related increase of brain resources shared by cognitive and motor control mechanisms (Montero-Odasso et al., 2019). This neural overlap would be reinforced with age by the increased cognitive monitoring required for motor control in order to compensate for the age-related decline of the sensorimotor system (Seidler et al., 2010). Motor control would then engage compensatory patterns of brain activity, most likely in the anterior brain regions, also known to be related to executive control (Li et al., 2018)

Recent investigations suggest that rather than linear, the interdependence between cognition and mobility could be a dynamic process that varies during aging (Best et al., 2016; Tian et al., 2017). However, little is known about the factors likely to affect this relationship. Cardiorespiratory fitness (CRF) could represent a potential moderator of the age-related increase of cognitive–motor association. CRF is a critical component of physical functions and reflects the cardiac and respiratory systems’ capacity to supply the exercise-induced muscular demand in oxygen (Ross et al., 2016). It is affected by a certain number of relatively well-determined factors. Some of them are nonmodifiable, such as sex, age, height, or genetic predisposition, and others are modifiable, such as weight and physical activity level, especially aerobic exercise training. In older adults, a greater CRF is associated with better executive functions (Colcombe & Kramer, 2003; Predovan et al., 2021) and mobility (Berryman et al., 2013; Fiser et al., 2010). However, little is known about its potential moderation of cognitive–motor relationships.

The present study aimed to investigate whether CRF moderates the effect of age on the association between executive functioning (EF) and mobility. A cross-sectional analysis, based on a three-way moderation model, addressed how the estimated CRF of individuals aged between 50 and 87 interacts with their age to moderate the relationship between EF and mobility. We expected that higher CRF would reduce the age-related increase of cognitive–motor interdependency.

## Research Design and Methods

### Participants

A total of 189 adults aged 50 and older performed baseline assessments of the COVEPIC (NCT04635462) and COVEPICARDIO (NCT04661189) studies. Participants (50–87) were divided into three groups according to their age: middle-aged (MA; 50–64), young older adults (YOA; 65–74), and old older adults (OOA; ≥75). Participant recruitment was performed by advising active members of the Montreal Heart Institute’s Centre for Preventive Medicine (Centre EPIC) about the project, by reaching out to community-dwelling adults through online communications, or by the Montreal Heart Institute’s (MHI) physicians. Individuals were included if they had access to a tablet (i.e., iPad or Android) or a computer with an Internet connection. Participants were excluded in case of significant cognitive impairment as characterized by a score of 19/23 or lower on the telephone version of Mini-Mental State Examination (Roccaforte et al., 1992), non-cardiopulmonary limitation to exercise (e.g., arthritis) or severe exercise intolerance, and respiratory disease (e.g., severe asthma, chronic obstructive pulmonary disease, and severe coronavirus disease 2019-related symptoms).

COVEPIC and COVEPICARDIO studies were conducted in compliance with International Conference on Harmonization Good Clinical Practice and all applicable regulatory requirements. Both received the approval of the MHI’s research ethics board (FWA00003235; ID: MHI 2019-2785). Participants’ consent was collected prior to assessments.

### Procedure

COVEPIC and COVEPICARDIO studies enrolled participants with and without cardiovascular disease (CVD), respectively. Their complete procedures were equivalent and were reported in their respective published protocols (Besnier et al., 2021; Dupuy et al., 2021). All assessments were performed remotely under videoconference supervision or using online questionnaires and were preceded by a technology tutorial aiming to ensure that participants’ Internet access and online tools were ready and sufficiently mastered. For the present study, only the data collected at baseline were used. Mobility, EF measurements, and CRF estimation were extracted from these data. The score obtained by participants in the remote version of the Montreal Cognitive Assessment (MoCA; Gagnon et al., 2022) and Physical Activity Scale for Elderly (PASE; Washburn et al., 1993) were also used to describe their global level of cognitive functioning and their level of physical activity.

#### Mobility assessment

Participants performed the Timed Up and Go (TUG) test remotely during the videoconference functional testing session to assess their mobility (Bohannon, 2006). To ensure an appropriate set-up for the TUG test at participants’ homes, a demonstration video of the test and the guidelines for functional testing, describing the space and material required, were sent 2 days before the session. At the beginning of the testing session, participants were required to put on a pair of usual comfortable flat shoes, and a screening of the testing set-up was done by the assessor using the camera and questioning the participant. For the TUG, the experimenter had to check the walking track with the participant and ensure that a measuring tape was used to fix its length. The experimenter also verified that the chair had a standard sitting height, armrests, and seat (e.g., no extra cushion), and was not an office chair with wheels. Participants started the TUG in a seated position and had to stand up, walk 3 meters at their usual pace, turn around, and then come back to the chair to regain the seated position. The stopwatch started when participants removed their back from the chair and stopped when they regained a sitting position. To guarantee the test’s reliability, the experimenter had to ensure the suitability of visual feedback, which had to include a clear view of the participants’ back on the chair. Three trials were performed, and the results are presented as their average.

#### Executive functioning assessment

Both phonologic verbal fluency and the Mental Alternation Test (MAT) were performed remotely during the videoconference neuropsychological testing session to assess EF (Delis et al., 2001; McComb et al., 2011). For the phonologic verbal fluency, participants were required to generate a maximum of words in 60 s with the initial letter “P” or “T” for the French- and English-speaking participants, respectively. Then, the number of words produced during the 60 s was noted to assess the performance. The MAT was performed in three steps: part A, part B, and part C. For parts A and B, participants respectively: counted from 1 to 20 and recited the alphabet while timed. The number of mistakes and time taken were noted, and participants had 30 s maximum to perform each task. For part C, participants were asked to name numbers and letters while switching between both and respecting chronological and alphabetical order, respectively (i.e., “1-A-2-B-3-C-4-D-5-E-6-F” …). Participants were timed for 30 s, and the number of switches and mistakes were noted. The total number of words produced during phonologic verbal fluency and the number of switches performed during the MAT part C were transformed into standardized *Z*-scores and averaged to provide a composite score of EF performance. A Cronbach’s α of 0.6 was found, indicating an acceptable internal consistency between the two variables (Bland & Altman, 1997).

#### Cardiorespiratory fitness estimation

To predict their CRF, participants had to fill out the Matthew’s questionnaire (Matthews et al., 1999). This questionnaire collected participants’ sex, age, anthropometric data (height and weight), and self-reported physical activity level to compute an estimation of their VO2 max in ml/kg/min. Matthew’s equation is a reliable and validated method for the cross-sectional estimation of VO2 max that is demonstrated to be strongly correlated with the direct measurement of CRF (i.e., standardized treadmill protocol; Peterman et al., 2020).

### Statistical Analysis

Analysis of variance (ANOVA) and Chi-squared tests were used to analyze the effect of the Group (MA, YOA, and OOA) on the participants’ characteristics, before running analysis of covariance (ANCOVA) analyses, adjusted for the relevant covariables, to assess the group effect on mobility, cognitive, and CRF outcomes. When significant, Bonferroni corrected post hoc tests were performed.

**Figure 1.**
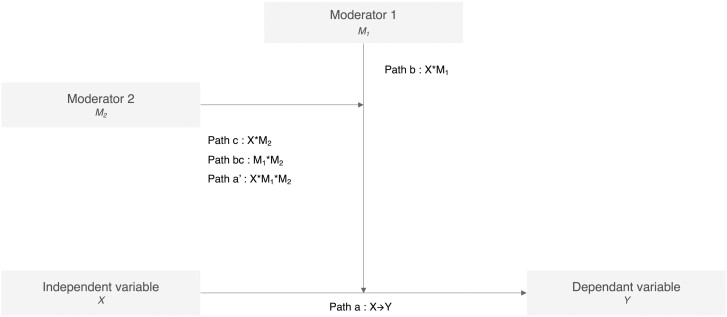
The three-way moderation model assessing the effect of age (M1) and cardiorespiratory fitness (M2) interaction on the relationship between executive functioning (X) and mobility (Y).

The association between EF and mobility in our sample was first investigated with a linear regression of EF (X) on TUG (Y), adjusted for age and the relevant covariables arising from the group characteristics’ comparison. Then, conditional process analysis addressed whether age interacts with physical fitness to moderate the relationship between EF and mobility. The PROCESS macro for SPSS was used to perform a three-way moderation analysis applying a series of logistic regressions to estimate the effects of the proposed moderator paths (Hayes, 2017; Figure 1). The model was built with numerical and categorical variables and adjusted for the relevant covariables. It included EF (*Z*-score) as the independent variable (X), TUG (seconds) as the dependent variable (Y), age group (MA; YOA; and OOA) as moderator 1 (M_1_), and estimated CRF (in ml/kg/min) as moderator 2 (M_2_). The proposed moderation model was tested in two steps. In the first step, the macro estimated the effect of the interaction between EF and the different moderators to predict TUG performance. In the second step, the model tested whether the effect of EF on TUG performance differed by age group and CRF level.

Power calculation was based on the effect size previously reported by Berryman et al. (2013) for the association between TUG and EF, using the flexibility score of the modified Stroop Color–Word test. In a regression model with seven predictors X, M_1_, M_2,_ and their interactions, a total sample size of 153 participants should yield 95% power to detect a medium effect size (*f*^2^ = 0.15). The centrality and normality of the residuals were verified. Neither heteroscedasticity nor multicollinearity was observed. All analyses were performed using SPSS statistics version 28 (IBM Corp, Armonk, NY), and the significance threshold for each test was set at 0.05.

## Results

### Age Group Characteristics

Table 1 synthetizes participant characteristics according to their age group (MA, YOA, and OOA). Chi-square tests showed that sex repartition differed between age groups (Chi^2^ = 28.31, *p* < .001), with a higher proportion of women in younger participants (69% of women in MA) and a higher proportion of men in older participants (85% of men in OOA). Age group did not directly affect CVD history. However, group × sex interaction showed that in our sample, female participants were more likely to be healthy (78% of the female participants), while male participants were more likely to have a CVD (73% of the men participants) with variation according to the age group. More precisely, in MA and YOA groups, female participants were, respectively, 4 and 3 times more likely to be healthy than male participants (MA: risk ratio [RR] = 4.32; Chi^2^ = 29.56, *p* < .001; YOA: RR = 3.11; Chi^2^ = 17.48, *p* < .001), whereas this difference remained a trend for the OOA group (OOA: RR = 2.24; Chi^2^ = 3.20, *p* = .065). Consequently, all the subsequent analyses, including comparative and regression-based statistics, were adjusted for sex and CVD.

**Table 1.** Comparison of Participants’ Characteristics According to Their Age Group

Variable	Total	Middle-aged	Young older adults	Old older adults	
	*N* = 189	*n* = 87	*n* = 69	*n* = 33	*p* Value
Demographic characteristics					^a^
Age (years)	65.85 (8.4)	58.65 (4.0)	69.04 (3.0)	78.59 (2.8)	**<.001** ^b^
Sex (% of women)	55.6%	69.8%	58.0%	15.2%	**<.001** ^c^
Education (years)	16.57 (3.4)	16.41 (3.3)	16.87 (3.6)	16.35 (3.4)	.967
Cardiovascular disease (% of individual)	45.0%	38.3%	46.4%	58.8%	.122
Body mass index (kg/m^2^)	28.19 (5.5)	28.24 (5.9)	28.67 (6.2)	27.10 (4.3)	.522
Mobility, cognition, and cardiorespiratory fitness					^d^
Physical activity scale for elderly (0–400)	135.61 (58.6)	143.65 (6.1)	137.58 (6.9)	110.50 (10.2)	**.023** ^b^
VO2 max (ml/min/kg)	25.85 (7.23)	28.03 (7.0)	23.9 (7.6)	24.1 (5.3)	**<.001** ^c^
Timed Up and Go (s)	8.22 (1.4)	7.98 (1.2)	8.30 (1.4)	8.65 (1.8)	.062
MoCA (0–30)	26.04 (2.3)	26.69 (1.9)	25.96 (2.2)	24.52 (2.6)	**<.001** ^c^ ^,^ ^e^
MAT part C (number of switches in 30 s)	26.51 (6.3)	26.72 (6.6)	26.48 (5.8)	25.61 (6.5)	.476
Phonologic verbal fluence (number of words in 60 s)	14.70 (4.0)	15.32 (4.0)	14.26 (3.7)	13.97 (4.1)	.119
Executive functioning *Z*-score	—	0.10 (0.9)	−0.06 (0.7)	−0.16 (0.9)	.178

*Notes*: MAT part C = Mental Alternation Test part C; MoCA = Montreal Cognitive Assessment; VO2 max = maximum volume of oxygen. All were reported in mean (*SD*) or %. ANOVA = analysis of variance; ANCOVA = analysis of covariance; CVD = cardiovascular disease; *SD* = standard deviation.

^a^The *p* values were calculated using ANOVA.

^b^ *p* Value < .05 for comparison between each age group.

^c^ *p* Value < .05 for comparison between old older adult and middle-aged groups.

^d^The *p* values were calculated using ANCOVA adjusted for sex and CVD.

^e^ *p* Values < .05 were highlighted in bold. *p* Value < .05 for comparison between old older adult and young older adult groups.

ANCOVAs were performed to investigate the group effect on mobility, cognition, and physical fitness. Results revealed a group effect on general cognitive functioning (*F*_2,184_ = 39.15, *p* < .001, η = 0.086), but no difference on EF outcomes (all ns). Post hoc comparisons showed that OOA obtained lower total MoCA scores than MA (mean diff. = −1.96; *p* < .001) and YOA (mean diff. = −1.28; *p* = .024). A trend for a group effect was also observed for TUG performance (*F*_2,184_ = 2.83, *p* = .062, η = 0.030), with OOA who tended to be slower on the TUG than MA (mean diff. = 0.70; *p* = .067). ANCOVAs also showed a group effect on CRF (*F*_2,184_ = 38.97, *p* < .001, η = 0.298), with a significant decrease of VO2 max through each age group (all *p* < .001). Finally, groups were also significantly different in their level of physical activity (*F*_2,184_ = 3.85, *p* = .023, η = 0.040). OOA obtained significantly lower PASE scores compared to MA (mean diff. = −34.99; *p* = .021), and tended to obtain lower scores compared to YOA (mean diff. = −30.17; *p* = .060).

### Regression-Based Analysis

The linear regression model, adjusted for age, sex, and CVD, explained 9.8% of the variance observed in TUG performance (*R*^2^ = 9.8; *p* < .001). It was found that EF significantly predicted TUG performance (β = −0.275, *t* = −2.292, *p* = .023), such that those with lower EF scores performed slower on the TUG.

**Figure 2.**
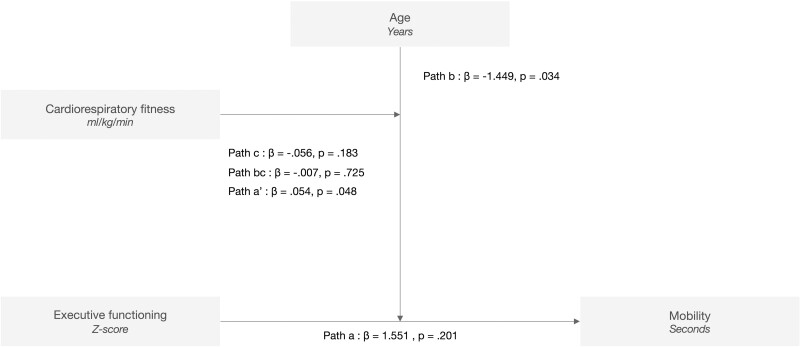
Interactions between executive functioning, age, and cardiorespiratory fitness, to predict mobility. Model: *R*^2^ = 17.6; *p* < .001.

Figure 2 shows the results of the three-way moderation model and reports the effect size for the different moderation pathways. The proposed model explained 17.6% of the variance observed in TUG performance (*R*^2^ = 17.6; *p* < .001). The model shows that the EF × age interaction significantly affects TUG prediction (β = −1.449; *p* = .034; Path b), confirming that the association between EF and TUG performance of participants differs according to their age group. The model also demonstrates that the EF × age × physical fitness interaction had a significant effect on TUG prediction (β = 0.054; *p* = .048; Path a’).

**Figure 3.**
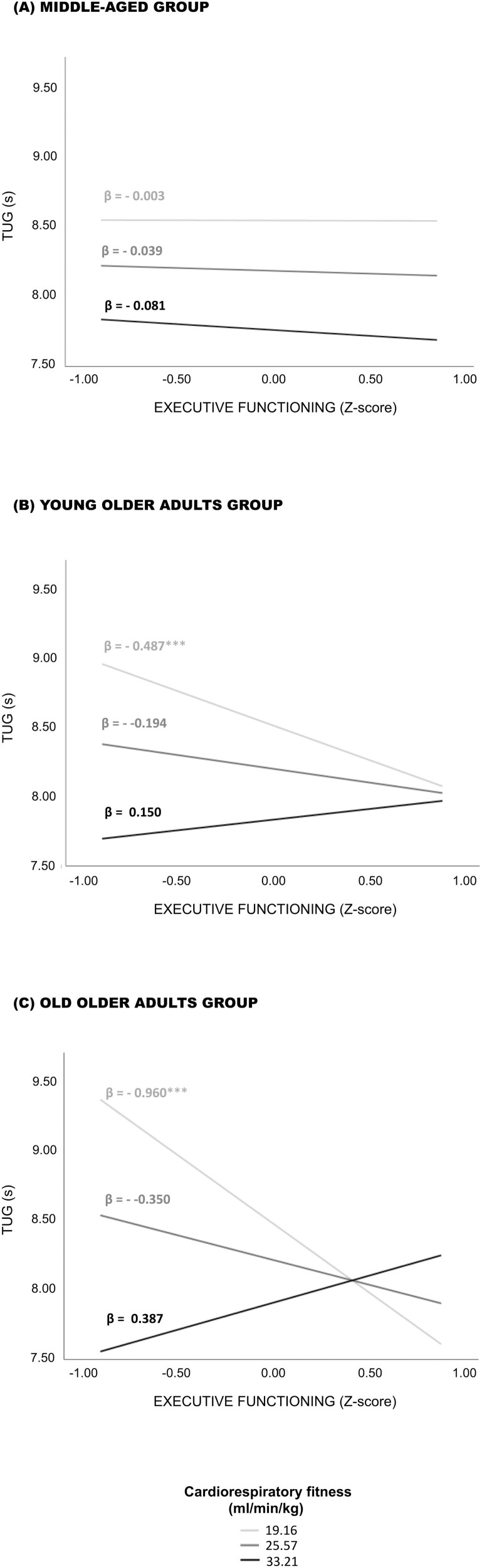
Association between executive functioning and TUG performance at determined values of cardiorespiratory fitness and age. TUG = Timed Up and Go. ***p < .005.

The conditional analysis showed that the TUG performance of older adults (i.e., YOA and OOA) with CRF estimates of 19.16 ml/min/kg or lower is positively associated with their EF performance. Each one-point increase on their EF *Z*-score (i.e., better performance) resulted in a 0.5 s decrease in TUG duration (i.e., faster performance) of lower fit YOA (β = −0.487; 95% confidence interval [CI95] = [−0.820; −0.154]; *p* = .004). A larger effect size was observed for lower fit OOA, with a decrease of 1 s of TUG duration (faster performance) per point earned on the EF *Z*-score (better performance; β = −0.960; CI95 = [−1.585; −0.356]; *p* = .002). When older adults’ CRF estimates were higher than 19.16 ml/min/kg, the association between EF and TUG became nonsignificant. No significant association between EF and TUG was observed for the MA group. These results are displayed in Figure 3.

## Discussion and Implications

This study addressed whether CRF moderates the age-related increase of cognitive–motor interdependency using a statistical model of moderated regression. In compliance with the literature, results first confirm that EF predicts mobility (Demnitz et al., 2016); here, the model explained 9.8% of the variance observed in participants’ TUG performance. Including age and CRF as moderators increased the proportion of variance explained by the model to 17.6% and confirmed that CRF interacts with age to moderate the association between EF and TUG performance. Results demonstrated that for a VO_2_ max estimated at 19.16 ml/min/kg or more, the interdependence between mobility and EF observed in older adults beyond 65 years disappears. The effect size of the association between EF and mobility increases after 75 years but again disappears at a VO_2_ max of 19.16 ml/min/kg or higher.

To the best of our knowledge, this study is the first to demonstrate that CRF is not only associated with improved mobility and executive functions but also directly affects the interdependence between mobility and cognition in older adults. Considering the accumulated knowledge from the motor control and neurocognitive aging researches (Li et al., 2018), one can expect that CRF affects brain resources shared by these two functions. Indeed, aerobic capacity is a strong predictor of older adults’ mobility, likely to affect the relative intensity of the fundamental components of daily life motor activity, such as walking (Fiser et al., 2010). With aging, maximal aerobic capacity naturally declines, slowly at first, with a rate of 3%–6% per decade, and more quickly thereafter, with a rate of 20% per decade beyond the age of 70 years (Fleg et al., 2005). According to the American College of Sports Medicine (ACSM) definition, for older adults walking at their usual speed represents a relatively vigorous exercise between 60% and 89% of their VO2 reserve (i.e., the difference between VO2_peak_ and VO2 during standing; ACSM, 2013). Indeed, it has been observed that walking at their usual speed represents almost 65% of the VO2 reserve for faster walkers older adults’, and even 87% or more for slower walkers (Fiser et al., 2010). Therefore, the relative intensity of older adults’ mobility is directly associated with their maximal aerobic capacity, but also with their functional capacity, both being interrelated. At the same time, the study conducted by Harada et al. (2009) brings convincing evidence that, in older adults, the cortical activity in the left prefrontal region is directly associated with the relative intensity of walking, as determined using the heart rate-based Karvonen method. More precisely, when older adults walk at a relative intensity of 70%, an increase of the left prefrontal cortex activity is observed. The intensity of this left prefrontal cortex engagement is directly correlated with individuals’ heart rate during the walk and is significantly influenced by their gait capacity. Therefore, the left prefrontal cortex, a region known to be involved in the executive process (Collette et al., 2006), demonstrates to also be involved in older adults’ mobility when its relative intensity exceeds 70%. In our study, a reasonable hypothesis could be that in older adults with lower aerobic capacity, mobility engages compensatory patterns of brain activity, involving prefrontal regions and thus strengthening the relationship between EF and mobility. Such compensatory brain activity could be directly related to the higher energy required for walking, but also indirectly to the muscular strength, balance, or sensory functions, crucially involved in mobility, assessed with the TUG, and likely to be less efficient in lower-fitness older adults (Li et al., 2018). Furthermore, a higher aerobic capacity is also known to be associated with improved brain functions (Colcombe & Kramer, 2003), which can reduce the relationship between executive function and mobility in higher-fitness older adults.

Physical activity, especially aerobic exercise, represents the major lever to maintain or improve CRF. In its recently published guidelines, the World Health Organization outlines the critical role of physical activity in maintaining older adults’ functional abilities and indicates that aerobic physical activity plays a significant role in reducing their risk of functional limitations and falls (Grade A: high level of evidence; Bull et al., 2020). Our observations converge towards the same conclusion and suggest that physical activity may affect the interdependence between mobility and EF through its impact on CRF. In this study, it is to note that a threshold of 19.16 ml/min/kg is observed for both YOA and OOA. However, depending on age and sex, a VO2 max beyond 19.16 ml/min/kg could be hard to achieve. If a VO2 max of 19.16 ml/min/kg refers to relatively low aerobic performance for a 65-year-old man, it represents the average and even high performance for a 75-year-old woman. From a clinical perspective, using a nomogram to estimate the percentage of predicted exercise capacity for a given sex and age could further inform the implication of this threshold for a given patient (Gulati et al., 2005). As expected, our data showed a lower level of physical activity in this age group. Hence, these observations support the current idea suggesting the added value of diversifying the approach to prevent age-associated loss of autonomy and personalize the proposed intervention, especially for OOA. Considering the stronger association between EF and the mobility of lower fit OOA, interventional approaches targeting the reinforcement of executive functions, such as cognitive training (Li et al., 2010; Marusic et al., 2018), could represent another interesting strategy to help improve and maintain mobility in older age. The postintervention results of COVEPIC and COVEPICARDIO studies (Besnier et al., 2021; Dupuy et al., 2021), investigating the effect of physical exercise alone and combined with cognitive training on cognitive and physical functions, will further address this question.

Despite interesting results, several limitations need to be considered to fully appreciate the scope of this study. First, considering its cross-sectional design, we cannot exclude the presence of confounding factors nor conclude on a potential causal relationships. Here, this also includes the unbalanced proportion of male and female participants between the age groups. All analyses were adjusted for the sex of participants to limit this bias. In addition, further investigations would be needed to determine to what extent the remote version of the TUG and verbal fluency tests are equivalent to their in-lab version. However, the present study provides some preliminary evidence concerning their potential validity. Indeed, the reported data fall within the ranges of reference for the in-lab tests (Bohannon, 2006; McComb et al., 2011; St-Hilaire et al., 2016), and the results show a remarkable consistency with previous reports regarding cognitive–motor aging (Demnitz et al., 2018; Donoghue et al., 2012). Also, the remote measurements developed for COVEPIC and COVEPICARDIO clinical trials are based on validated methods (Bohannon, 2006; McComb et al., 2011; St-Hilaire et al., 2016) and, when available, follow published recommendations for the remote assessment (Madhavan et al., 2021). In the same vein, nonexercise-based prediction can lead to a loss of precision in estimating CRF, especially in quantifying intervention-related change (Peterman et al., 2020) or assessing its association with cognition (Predovan et al., 2021). Additional exploration using an in-lab maximal exercise protocol and separate analyses for male and female participants could thus be particularly relevant to document the phenomenon further and produce a more reliable and precise VO2 max threshold.

In conclusion, this study provides evidence of a dynamic relationship between mobility and EF over aging and demonstrates that a higher CRF reduces their interdependency in older adults. These results are a starting point for future investigations aiming to confirm the observations and shed light on the underlying mechanisms.
